# 91. Implementation of a blood culture algorithm in emergency department patients as a diagnostic stewardship intervention

**DOI:** 10.1093/ofid/ofad500.007

**Published:** 2023-11-27

**Authors:** Jessica Seidelman, Rebecca Theophanous, John Ramos, Alyssa R Calland, Rachel Krcmar, Priya Shah, Lucas Tarabal da matta, Stephen Shaheen, Rebekah Wrenn

**Affiliations:** Duke University School of Medicine, Durham, NC; Duke University School of Medicine, Durham, NC; Duke University Hospital, Durham, North Carolina; Duke University, Durham, North Carolina; Duke University Hospital, Durham, North Carolina; Duke University Hospital, Durham, North Carolina; Duke University, Durham, North Carolina; Duke University Medical Center, Durham, North Carolina; Duke University, Durham, North Carolina

## Abstract

**Background:**

Blood cultures (BCx) are commonly ordered for patients at low risk of bacteremia. Liberal ordering can increase false-positive results due to contamination, along with increasing length of stay, excess antibiotics, and unnecessary diagnostic procedures. We implemented an algorithm for appropriate blood culture obtainment (Figure 1) in an academic tertiary care emergency department (ED) and assessed the intervention’s impact on various operational, clinical, and safety metrics.

**Figure 1:**
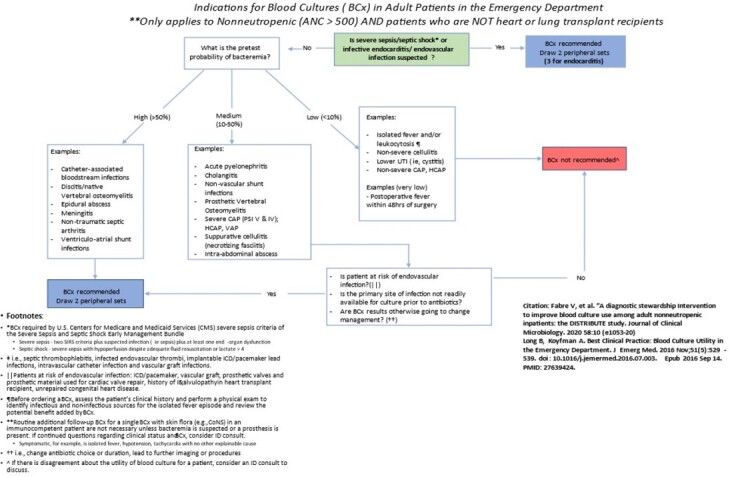
ED Blood culture algorithm

**Methods:**

We performed a prospective cohort study in the Duke University Hospital ED from 12/2022 to 3/2023 using historical controls from 12/2020-11/2022. The BCx algorithm was disseminated in-person, electronically, and accessible online. Weekly chart review of all eligible patients for algorithm adherence was completed by 7 ED clinicians with monthly feedback provided to all ED providers. We defined a BCx event as 1 or more BCx sets within 24 hours. We excluded patients < 18 years, absolute neutrophil count < 500 10^9^/L, and heart and lung transplant recipients. We measured BCx order volume, indication, algorithm adherence, BCx positivity rate, antibiotic days of therapy (DOT), and 30-day hospital readmission.

**Results:**

Average monthly BCx volume was 1387 pre-intervention compared to 1226 post-intervention (p= 0.046). An increase in culture positivity was see post-intervention with a rate of 13.9% compared to 11.23% in the pre-intervention group (Figure 2, p< 0.001). The majority of the 2168 BCx adhered to the algorithm. (Figure 3). The most common reasons for non-adherence were isolated fever or leukocytosis (29%), non-severe community-acquired pneumonia or healthcare-associated pneumonia (6.9%) and non-severe cellulitis (4%). No change in DOT (786 vs. 783 days of therapy per 1000 patient days, p-value 0.85) (Figure 4) or monthly 30-day hospital readmission (21.1% vs 19.6%, p-value 0.30) was observed.

**Figure 2:**
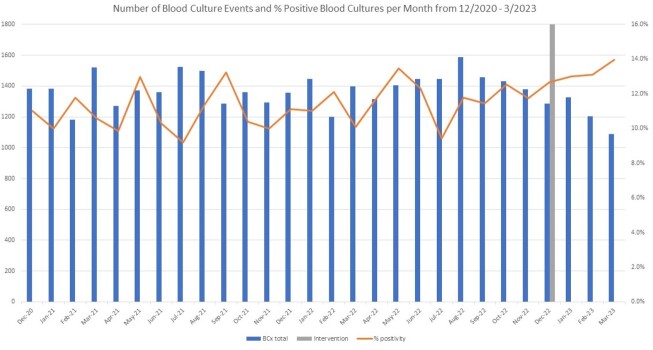
Blood cultures drawn per month and % appropriate pre/post-intervention (N=2168)

**Figure 3:**
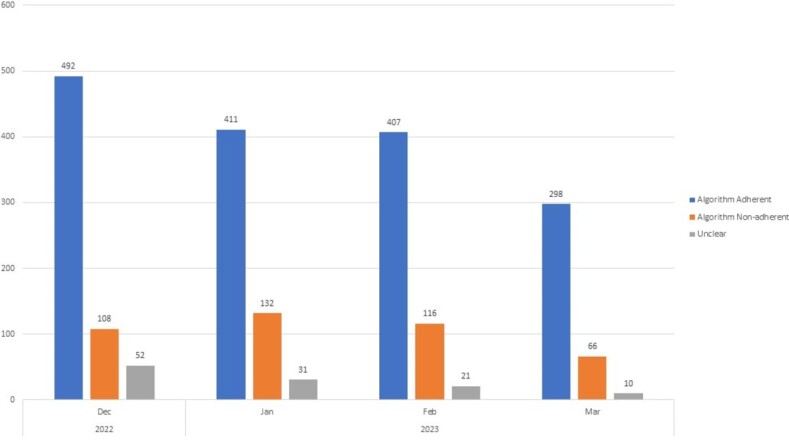
Algorithm Adherent vs. Algorithm Non-Adherent blood cultures per month (N=2168)

**Figure 4:**
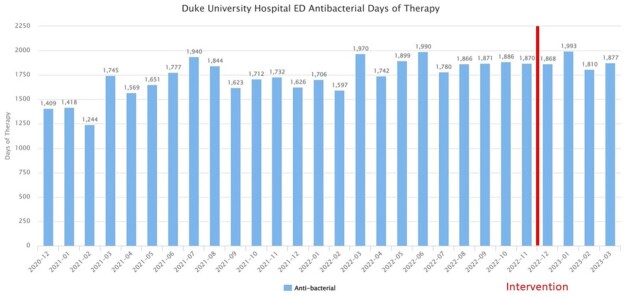
Emergency Department anti-bacterial days of antibiotic therapy per 1000 patient days

**Conclusion:**

Introduction of a BCx algorithm in an academic tertiary care ED, resulted in a decrease in BCx volume, increase in BCx positivity rate, and no increase in DOT or readmission was observed. (Table 1)

**Table 1:**
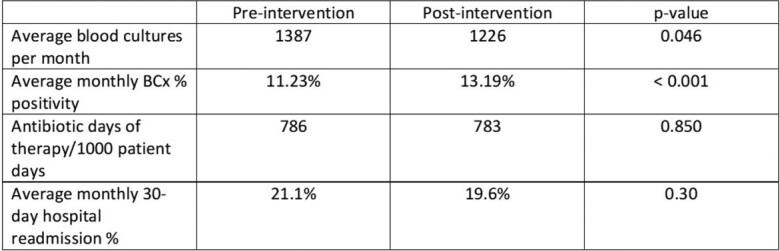
Blood culture volume, positivity rate, days of antibiotic therapy, and 30-day readmission

**Disclosures:**

**Jessica Seidelman, MD, MPH**, Uptodate: content editor for pelvic osteomyelitis page

